# *In Vivo* Screening for Anti-Osteoporotic Fraction from Extract of Herbal Formula Xianlinggubao in Ovariectomized Mice

**DOI:** 10.1371/journal.pone.0118184

**Published:** 2015-02-19

**Authors:** Xinluan Wang, Yixin He, Baosheng Guo, Man-Ching Tsang, Fengjuan Tu, Yi Dai, Zhihong Yao, Lizhen Zheng, Xinhui Xie, Nan Wang, Xinsheng Yao, Ge Zhang, Ling Qin

**Affiliations:** 1 Translational Medicine R&D Center, Institute of Biomedical Engineering and Health Tec, Shenzhen Institutes of Advanced Technology, Chinese Academy of Sciences, Shenzhen, 518000, China; 2 Musculoskeletal Research Laboratory, Department of Orthopaedics & Traumatology, The Chinese University of Hong Kong, Hong Kong SAR, China; 3 Institute of Traditional Chinese Medicine & Natural Products, College of Pharmacy, Jinan University, Guangzhou, 510632, China; Shanghai University of Traditional Chinese Medicine, CHINA

## Abstract

**Background and Objectives:**

Traditional Chinese Medicine (TCM) Fufang or formula Xianlinggubao (XLGB) is a prescribed TCM drug in China registered for prevention and treatment of osteoporosis. Fufang in TCM is comprised of a group of herbal compounds contributing in group to the treatment efficacy. The present study aims to identify the bioactive fraction(s) in XLGB extract that account(s) dominantly for its osteogenic effects.

**Methods:**

The extract of XLGB formula was separated into three fractions using chromatography, i.e., XLGB-A, XLGB-B and XLGB-C. They were administrated to 4-month old ovariectomized (OVX) mice for 6 weeks to determine which bioactive fraction(s) were more effective for preventing OVX-induced bone loss evaluated by microCT, biomechanical testing and biochemical markers. The main peaks of the key fraction were identified using reference compounds isolated from the fraction. In addition, the effects of the composite compounds in XLGB-B on osteoblasts’ proliferation and mineralization were evaluated in UMR 106 cells.

**Results:**

XLGB-B with a yield of 13.0% from herbal Fufang XLGB was identified as the most potential one among the three fractions for prevention of OVX-induced bone loss confirmed with bone mass, bone microarchitecture, bone strength and bone turnover markers. Nine compounds in HPLC fingerprint were identified in the XLGB-B fraction, including phenylpropanoids from *Herba Epimedii*, terpenes from *Radix Dipsaci* and coumarins from *Fructus Psoraleae*. In addition, the identified compounds effectively promoted proliferation and/or mineralization of osteoblast-like UMR 106 cells *in vitro*.

**Conclusion:**

XLGB-B with defined phytochemical structures was screened as the key fraction that demonstrated preventive effects on OVX-induced bone loss in mice. The present study laid down a foundation towards a new generation of herbal Fufang characterized with “less herbal materials for achieving equal treatment efficacy” in development strategy of TCM for prevention of OVX-induced osteoporosis.

## Introduction

Osteoporosis is a systemic disorder characterized with low bone mass and deterioration of bone micro-architecture, and a consequent increase in bone fragility and fracture risk[1,2]. With a worldwide increase in aging populations, osteoporosis has become a serious global health problem. Prevention of osteoporosis is essential to avoid osteoporotic fractures, especially in females after onset of menopause. Hormone (estrogen) replacement therapy (HRT) is the first-line therapy for prevention of postmenopausal bone loss[3]. However, after the release of the Women’s Health Initiative (WHI) trial data, concerns are raised among both postmenopausal women and their physicians due to the increased risk of breast cancer and cardiovascular events[4]. In China, therapy with herbal Fufang is a popular alternative in traditional Chinese Medicine (TCM) developed for prevention and treatment of osteoporosis and related bone diseases. A class of plant-derived substances, the so-called “phytoestrogen” has been identified from TCM and proven possessing anti-osteoporotic effects yet without resulting in estrogen-associated adverse effects, such as stimulation of reproductive organs, reported in both experimental studies [5–7] and clinical trials [8,9].

Xianlinggubao formula (XLGB) was formed based on modification of the empirical “Miao minority” medicine, which was commonly used to tone the “kidney system” and nourish bones [10]. XLGB capsule was officially approved by the Chinese State Food and Drug Administration (CFDA) as the over-the-counter (OTC) drug for treatment of osteoporosis, osteoarthritis, aseptic osteonecrosis and bone fracture [9]. As a phytoestrogen-rich TCM [11], preclinical studies showed that XLGB improved BMD and mechanical strength in ovariectomized (OVX) rats model [6,12,13] and prevented OVX-induced deterioration of hip in old rats [6]. Clinical data demonstrated its positive effects in promoting fracture healing [14] and treatment of osteoporosis [8,15]. Recently, a multicenter, double blind, placebo-controlled, and dose-effect clinical trial further confirmed the safety and efficacy of XLGB in postmenopausal women with osteoporosis [9].

As herbal Fufang, XLGB consists of six herbs with percentages in weight as follows: *Herba epimedii* (70%), *Radix dipsaci* (10%), *Radix salvia miltiorrhizae* (5%), *Rhizoma anemarrahenae* (5%), *Fructus Psoraleae* (5%), and *Rehmannia glutinosa* (5%)[9]. Chemical analysis showed that there are a large amount of compounds with diversity of structures in this formula, such as, flavonoids [11], coumarins [16], saponins [17], alkalines [18], sugars [19], and terpenes [20]. So far, many reports on XLGB were pertinent to clinical observations [14,21,22], but research on the relationship between active compounds and its efficacy on preventing osteoporosis was lacking. Numerous compounds found in herbal formula made it difficult to conclude which compound(s) contributed more to the treatment efficacy. Nevertheless, it was feasible and clinically relevant to identify the most bioactive fraction(s) in Fufang[23], as such approach could prepare a herbal drug with fewer chemical constituents, i.e. with less material amount for encapsulation and convenient use so as improvement of drug compliance. In order to elaborate the key constituents from the clinically available XLGB for preventing bone loss, this *in vivo* screening efficacy study was designed to identify essential anti-osteoporotic fraction(s) from extract of XLGB Fufang using ovariectomized (OVX) mice and further confirmation of relevant bioactive compounds with osteogenic potential *in vitro*.

## Materials and Methods

### Preparation of XLGB extract and its sub-fractions[23]

Raw medical powder of XLGB capsule (Lot no 080371, 800 g) provided by Guizhou Tongjitang Pharmaceutical Co., Ltd. was soaked with 60% ethanol (8 L) overnight and then reflux extracted twice, 2 hrs for each time. Extracting solution was filtered and the filtrate was concentrated for removing ethanol, Followed by adding distilled water to a constant volume to 5000 mL. The well-mixed diluent was freeze-dried to obtain dry extract of XLGB (518.1 g) and the yield is 64.8%.

450 g of XLGB extract was suspended in 5 L of distilled water and centrifuged for 30 min at 3000 rpm, the supernate was chromatographed on a HP-20 macroporous resin column (*φ* 9 × 70 cm) and eluted with gradient of water, 30% (V/V) and 95% (V/V) alcohol to give XLGB-A (227.2 g), XLGB-B (61.3 g) and XLGB-C (109.3 g), respectively. The summary of the yield of each fraction was in Table 1.

**Table 1:** Fractions of XLGB* and their yield.

Fraction	Isolated solvent (v/v)		Isolated content (g)	Percentage of total crude extract (%)
	Ethanol (%)	Water (%)		
XLGB-A	0	100	227.2	58.9
XLGB-B	30	70	61.3	13.0
XLGB-C	95	5	109.3	23.2

*450 g of XLGB extract was chromatographed on a HP-20 macroporous resin column (*φ* 9 × 70 cm) and eluted with water and ethanol in gradient to give its fractions.

### Animals, grouping, treatment, and sampling

Four-month-old female C57/BL6 mice (body weight 22.3 ± 2.6 g) were used and Animal Experimentation Ethics Committee of the Chinese University of Hong Kong approved the care and experimental protocol of this study (Ref No. 07/068/MIS).

**Selection of optimal dose.** The mice were either sham-operated (Sham, n = 10) or ovaritecomized (OVX, n = 40). The number of the sample size was calculated based on our previously published studies [24]. All of the mice were weighted, and then divided into different groups randomized by using the random function in excels. The OVX mice were randomly assigned into the following four groups: OVX group, and three treatment groups with low, middle and high doses, i.e. XLGB-L, -M, and –H groups (OVX mice with 118 mg, 236 mg and 472 mg XLGB extract/kg body weight/day, respectively). Both the Sham and OVX mice were treated with saline, the vehicle used to suspend XLGB extract and fractions. In our previous clinical study [9], it was found that the effects of the middle dose (clinical prescription dose) was better than the higher one (double of the middle dose), so we choose corresponding clinical prescription dose to be equivalent to the high experiment dose, and the dose for the mice was calculated according to the Human-Mice Equivalent Dose Conversion Principle [25].

**Screening of active fraction(s) of XLGB.** The mice were either sham-operated (Sham, n = 10) or ovaritecomised (OVX, n = 50). The OVX mice were randomly assigned into the following four groups: OVX group (treated with vehicle), XLGB-A group (treated with 140 mg XLGB-A/kg body weight/day), XLGB-B group (treated with 31 mg XLGB-B/kg body weight/day), XLGB-C group (treated with 55 mg XLGB-C/kg body weight/day), and combined XLGB group (Treated with 140 mg XLGB-A, 31 mg XLGB-B and 55 mg XLGB-C/kg body weight/day). The experimental doses for the fractions of XLGB extract in the design were deduced from the results of the optimized dose determined above and the yield of each fraction.

Dose of the fraction = Optimized Dose of the extract×yield of the fraction

Vehicle, XLGB extract and fractions were all administrated orally through a custom-made stomach tube, which started on the day 4 after OVX for 6 weeks.

**Sample harvesting.** All mice were injected intraperitoneally with xylenol orange (30mg/kg) and calcein green (10mg/kg) in a time sequence of 10 and 2 days before euthanasia for studying bone mineral apposition[26]. After laparotomy using ketamine and xylazine (intraperitoneally, 100 mg/kg body weight and 4 mg/kg body weight, respectively) at the end of 6-week treatment, blood sample was collected via abdominal aorta puncture for serum isolation. Serum samples were then stored at -80°C for assaying the biomarkers. After euthanizing the animals proximal tibiae and vertebrae were dissected for measurement of trabecular micro-architecture; the left femur was subject to three-point bending test, and the right one was collected for bone histomorphometric analysis.

### Micro-computed tomography (MicroCT) analysis

The proximal tibia and the fifth lumbar vertebra were scanned by microCT system (viva-CT40; Scanco Medical, Switzerland), with a voxel size of 16.4 mm. 3-D reconstructed vertebra was used for histomorphometric analysis. For proximal tibia 80 continuous slices beginning at 0.3 mm from the most distal aspect of the growth plat extending distally along the tibia diaphysis were selected for analysis. All the trabecular bone from each selected slice was segmented for 3-D reconstruction (Sigma = 1.2, Supports = 2 and Threshold = 220) to calculate bone mineral density (BMD), bone volume/tissue volume (BV/TV), trabecular number (Tb.N), trabecular thickness (Tb.Th), and trabecular separation (Tb.Sp) [26].

### Mechanical testing

A material test machine (H25KS; Hounsfield Test Equipment Ltd. UK) with a 25 N load cell was used for three-point-bending test. The left femora were positioned horizontally with the anterior surface upwards, centered on the supports with 10 mm apart. A displacement rate of 5 mm/min was selected for applying the loading vertically to mid-shaft with anterior surface upward using our established protocol [24]. Failure force (N) was recorded for statistical comparison.

### Dynamic bone histomorphometric analysis

The right femur from mice was dehydrated in graded concentrations of ethanol and embedded without decalcification in modified methyl methacrylate. Frontal sections for trabecular bone were prepared for the distal femur at a thickness of 10μm (Jung Supercut 2065 microtome, Leica Microsystems, Germany). Trabecular sections from the distal femora were used for evaluating bone formation rate/bone surface (BFR/BS) using a professional image analysis software (ImageJ, NIH, USA) under fluorescence microscope (Leica image analysis system, Q500MC, Germany) [26,27]. The bone histomorphometric parameters were calculated and expressed according to published guideline for bone histomorphometry [28]. In details, the mean distance between two sequential fluorescent lines was calculated by the formula of area between two labeling divided by the mean perimeter of the labeling using Image J software.

### Analysis of bone turnover markers

Bone turnover markers in the serum, procollagen type I N-terminal propeptide (PINP) (bone formation marker) and C-terminal telopeptide of type I collegen (CTX) (bone resorption marker), were assayed by using ELISA kits (IDS Ltd., Boldon, UK) [26]. Briefly, 50 μl serum was added to the polyclonal rabbit anti-PINP or anti-CTX coated plate and incubated with 50 μl biotin labeled PINP or CTX for 1 hr. Then 150 μl avidin linked horseradish peroxidase was added to each well and incubated for 30 mins. Next, 150μl tetramethylbenzidine (TMB) substrate was added for color development and finally it was measured by using a microplate reader within 30 mins after adding the stop solution. Six calibrators at different PINP or CTX concentrations supplied by the company were measured for the standard curve. Two samples provided by the supplier were taken as the positive control.

### Chromatographic conditions for XLGB-B fingerprint profiles

The fingerprint of XLGB-B was established using the Agilent series 1200 HPLC system (Agilent Technologies, Santa Clara, CA, USA). The bioactive fraction was subjected by a reversed-phase (RP)-HPLC column with a gradient of water (A: 0.1% acetic acid)-acetonitrile (B: 0.1% acetic acid) as the mobile phase (0–5 min 2%B,80 min 36%B,95 min 48%B,110 min 80%B,112–125 min 100%B), at a flow rate of 0.8 ml/min and the column temperature was at 35°C. Detection wavelength was at 270 nm in the ultra-violet (UV) detector, and the drift tube temperature for Evaporative Light-scattering Detector (ELSD) was set at 115°C and the nitrogen flow-rate was of 3.2 L/min. The chromatographic peaks were identified by comparing their retention time with that of each reference compound, which was eluted in parallel with a series of mobile phases. In addition, spiking samples with the reference compounds isolated from XLGB-B further confirmed the identities of the peaks.

### *In vitro* study of identified compounds in XLGB-B fingerprint profiles

The UMR106 cell line (CRL-1661) and 3T3-L1 preadipocytes (CL-173) were purchased from the American Type Culture Collection (ATCC) and cultured according to our previously published protocol [23].

**Colorimetric MTT (Tetrazolium) assay for cell proliferation.** UMR 106 cells were placed into 96-well plate and maintained for 24 hr and then treated with the identified compounds (peak 1 to 9) at the concentrations of 0, 10, 100, and 1000 nM. MTT solution with the concentration of 1 mg/mL was added to each well at 37°C after 24 hrs treatement. After 4 hrs incubation, MTT was removed and 200 μL DMSO was added into all wells. The plates were then read using a micro-plate reader system (Microplate Spectro, Biotek Instrument Inc, USA) with a test wavelength of 570 nm against a reference wavelength of 650 nm [29].

**Calcium Nodules formation.** Osteogenic medium (10 mM β-Glycerophosphate, 50 μg/ml ascorbic acid) with solvent or xanthogalenol was used for osteogenic in UMR 106 cells. The cells were cultured for 48 hrs to reach confluence, and then the osteogenic medium with 100 nM of identified compounds was added. After culturing for 5 days, Alizarin Red S staining was performed to visualize calcium nodules formation using standard protocols [30]. Pictures of Alizarin Red S staining were scanned using Epson Perfection 4990 Photo Scanner (Epson America. Inc., USA). The results of calcium nodules staining were analyzed using Image J 1.32j (NIH, USA).

**Adipocyte differentiation and Oil Red O staining in 3T3-L1 cells.** For adipogenesis, 3T3-L1 cells (5 × 10^4^ cells/well) were plated into a 6-well plate and maintained for 2 days after reaching confluence (designated as day 0). Media were exchanged with differentiation medium (DMEM containing 10% FBS, 0.5 mM IBMX, 1 μM dexamethasone, 2 μg/mL insulin, and 200 μM indomethacin) for 2 days. The cells were then incubated in adipocyte growth medium (DMEM supplemented with 10% FBS and 1 μg/mL insulin) until day 8. Compounds (1 μM) and vehicle DMSO were added into the medium over the full course of differentiation. Medium was changed every other day. On day 8, the cells were stained with Oil Red O staining, an indicator of cell lipid content, and digitalized by a Leica microscope DMI3009B (Germany) form analysis [31].

### Statistical analysis

All data were expressed as mean ± standard deviation (SD) and analyzed by one way analysis of variance (ANOVA) with Bonferroni *post hoc* test to determine group differences. The analyses were performed using a statistical software program (SPSS version 17.0, SPSS, Chicago, IL, USA). *P* < 0.05 was considered to be statistically significant. The sample size of n = 10 was used for micro CT, mechanical testing, bone histomorphometric analysis and biochemical marker analysis. The proliferation and adipogenesis experiments were performed with at least 3 independent experiments, and the analysis was carried out with triplicate samples.

## Results

### MicroCT analysis for choosing the optimal dose of XLGB extract

As shown in Table 2, OVX mice had significantly lower BMD, BV/TV and Tb.Th in the sixth lumbar vertebrae when compared to the Sham group (*P* < 0.01). The decrease in BMD and BV/TV induced by OVX in mice were reversed significantly in XLGB-M group (*P*<0.05 in BMD and *P*<0.01 in BV/TV, *vs* OVX), while OVX mice in XLGB-L and XLGB-H group also prevented the decrease in BMD and trabecular bone microstructure to a certain extent, but with no significant difference as compared with OVX group. Therefore, middle dose of XLGB was selected as an optimal dose for further study as specified below.

**Table 2:** Effects of three doses of XLGB extracts on bone parameters as measured by microCT at the sixth lumbar vertebrae in OVX mice (mean values ± standard deviations, n = 10).

Treatment#	BMD(mgHA/cm^3^)	BV/TV(%)	Tb.Th(mm)
OVX	207.32±19.08	16.29±4.05	0.0753±0.0053
Sham	245.28±15.50**	24.28±2.30**	0.0895±0.0043**
XLGB-L	220.82±8.57	19.12±1.61	0.0779±0.0021
XLGB-M	228.44±12.26*	20.78±2.59**	0.0784±0.0041
XLGB-H	220.20±13.51	19.17±2.66	0.0767±0.0025

BMD: bone mineral density; BV/TV: bone volume/tissue volume; Tb.Th: trabecular thickness; Sham: sham-operated; OVX: ovariectomized; XLGB-L: OVX mice treated with 118 mg XLGB extract/kg body weight/day; XLGB-M: OVX mice treated with 236 mg XLGB extract/kg body weight/day; XLGB-H: OVX mice treated with 472 mg XLGB extract/kg body weight/day.

Mean value was significantly different from that of the OVX group

* P<0.05

** P<0.01

#Mice were subjected to the treatment for 6 weeks.

### MicroCT evaluation of proximal tibiae and the sixth lumbar vertebrae on the effects of XLGB fractions

As shown in Table 3, OVX significantly reduced BMD and BV/TV at the sixth lumbar vertebrae (*P*<0.01 *vs* Sham) and the proximal metaphysis of tibia (*P*<0.01 *vs* Sham) in mice. XLGB-B and combined XLGB groups showed excellent effects on prevention of BMD decrease (*P*<0.05 *vs* OVX), BV/TV (*P*<0.05 *vs* OVX) and Tb.Th (*P*<0.05 *vs* OVX) in the sixth lumbar vertebrae as compared with XLGB-A and XLGB-C. The decrease in BMD and the deterioration of bone microstructure at the proximal tibia induced by OVX was reversed significantly in all three groups, including XLGB-B group (*P*<0.01 in BMD and BV/TV, *P*<0.05 in Tb.N, Tb,Th and Th.Sp) or XLGB-A group (*P*<0.01 in BMD and BV/TV, *P*<0.05 in Tb.N) or XLGB-C group (*P*<0.05 in BMD, *P*<0.05 in BV/TV and Tb.N) or combined XLGB group (*P*<0.01 in BMD and BV/TV, *P*<0.05 in Tb.N).

**Table 3:** Effects of XLGB fractions on bone parameters as measured by microCT at the sixth lumbar vertebrae and proximal tibia in OVX mice.

Treatment#	The 6th Lumbar Vertebrae					Proximal Tibia				
	BMD(mgHA/cm^3^)	BV/TV(%)	Tb.N(1/mm)	Tb.Th (mm)	Tb.Sp(mm)	BMD(mgHA/cm^3^)	BV/TV(%)	Tb.N(1/mm)	Tb.Th (mm)	Tb.Sp(mm)
OVX	198.48±20.46	18.3±2.54	3.62±0.36	0.058±0.0042	0.288±0.0294	29.40±9.07	3.43±0.37	0.0471±0.0061	2.23±0.22	0.4591±0.0509
Sham	226.78±15.66**	22.2±2.38**	3.87±0.26	0.064±0.0053**	0.269±0.0183	48.70±12.72**	4.43±0.62**	0.0490±0.0025	2.24±0.23	0.4610±0.0453
XLGB-A	194.95±21.44	18.3±2.71	3.68±0.39	0.056±0.0052	0.285±0.0299	49.16±5.60**	5.03±0.97**	0.0531±0.0063*	2.44±0.22	0.4153±0.0376
XLGB-B	217.08±22.76*	21.4±3.16*	3.95±0.51	0.063±0.0067*	0.267±0.0395	53.37±8.66**	5.27±0.67**	0.0540±0.0073*	2.49±0.30*	0.4020±0.0392*
XLGB-C	201.67±22.84	18.9±3.36	3.42±0.41	0.060±0.0053	0.309±0.0405	39.88±13.14*	4.46±0.96**	0.0551±0.0072**	2.19±0.27	0.4653±0.0627
Combined XLGB	212.85±22.37*	20.1±3.03	3.63±0.55	0.062±0.0042	0.293±0.0427	45.00±11.90**	4.90±1.01**	0.0534±0.0026*	2.33±0.29	0.4322±0.0776

BMD: bone mineral density; BV/TV: bone volume/tissue volume; Tb.N: trabecular bone number; Tb.Th: trabecular thickness; Tb.Sp: trabecular bone separation; Sham: sham-operated; OVX: ovariectomized; XLGB-A: OVX mice treated with 140 mg XLGB-A/kg body weight/day; XLGB-B: OVX mice treated with 31 mg XLGB-B/kg body weight/day; XLGB-C: OVX mice treated with 55 mg XLGB-C/kg body weight/day; Combined XLGB: OVX mice treated with 140 mg XLGB-A, 31 mg XLGB-B and 55 mg XLGB-C kg body weight/day.

Mean value was significantly different from that of the OVX group

* P<0.05

** P<0.01

#: Mice treated for 6 weeks.

(mean values ± standard deviations, n = 10).

### Mechanical testing for OVX mice treated with different XLGB fractions

As shown in Table 4, maximum load and stiffness of the left femur mid-shaft were significantly higher in OVX mice in response to treatment with XLGB-B fraction (*P*<0.05 in maximum load and *P*<0.01 in stiffness), while the stiffness was significantly higher in the combined XLGB (*P*<0.05 in stiffness), but XLGB-A and XLGB-C did not show such effects on the maximum load and stiffness. However, all of the fractions had no significant effects on changes of energy of breaking.

**Table 4:** Effects of XLGB fractions on biomechanical properties, bone apposition rate and bone turnover marker in OVX mice (mean values ± standard deviations, n = 10).

Treatment#	Maximum load (N)	Energy (×10^−3^ J)	Stiffness (N/mm)	FBR/BS(μm^3^/μm^2^/d)	PINP (ng/mL)	CTX (ng/mL)
OVX	15.19±2.04	3.37±2.64	45.82±4.70	1.21±0.20	8.87±2.64	30.89±13.12
Sham	18.49±0.57*	4.2±1.18	58.71±3.99**	0.73±0.094**	6.00±1.18**	23.76±7.157
XLGB-A	17.75±1.23	3.467±1.38	53.28±2.22	1.07±0.13	8.98±1.38	25.52±12.76
XLGB-B	18.35±2.28*	4.15±1.82	56.53±3.02**	0.86±0.13**	6.77±1.82*	21.35±3.78*
XLGB-C	17.48±1.00	3.8±2.06	51.72±4.22	1.10±0.20	9.17±2.06	44.07±13.94*
Combined XLGB	17.45±1.41	3.6±0.78	54.54±4.98*	0.82±0.12**	6.95±1.24*	24.59±7.54

Sham: sham-operated; OVX: ovariectomized; XLGB-A, OVX mice treated with 140 mg XLGB-A/kg body weight/day; XLGB-B, OVX mice treated with 31 mg XLGB-B/kg body weight/day; XLGB-C, OVX mice treated with 55 mg XLGB-C/kg body weight/day; Combined XLGB: OVX mice treated with 140 mg XLGB-A, 31 mg XLGB-B and 55 mg XLGB-C kg body weight/day.

Mean value was significantly different from that of the OVX group

* P<0.05

** P<0.01

#: Mice treated for 6 weeks.

### Two-dimensional hisomorphometry of OVX mice treated with different XLGB fractions

**Fig 1:**
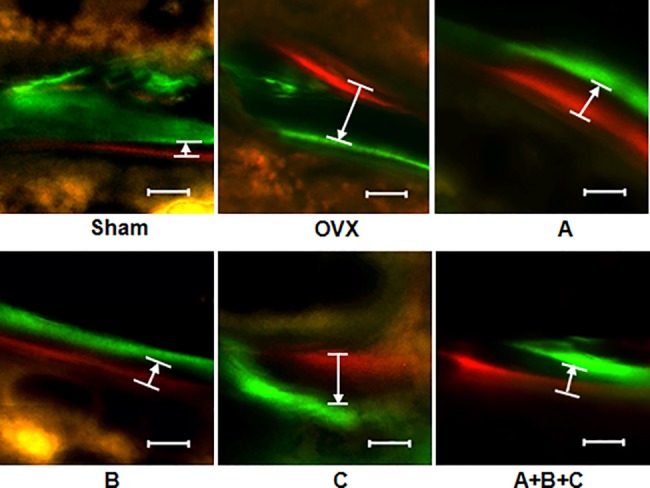
Representative fluorescent micrographs of the trabecular bone sections show the apposition of the xylenol (red) and calcein (green) labels in the sham-operated (Sham), ovariectomized only (vehicle-treated) (OVX), and OVX mice treated with different XLGB fractions. The white arrow pointed to the labeled the distance between two sequential fluorescent dyes in a time interval of 8 days. 20 x, bar = 10um.

The bone histomorphometry analysis showed that the bone formation rate (BFR/BS) of OVX mice was higher than that of the Sham group, indicating that the bone turnover increased after OVX. BFR/BS in XLGB-B and combined XLGB group was significantly lower compared to that of OVX group (*P*<0.05), whereas the other groups had moderately bot not statistically significant lower bone apposition rate (**Table 4**). Representative pictures demonstrated that extensive xlyenol and calcein labeling of trabecular surfaces with narrow width between the two dyes in the mice treatment with XLGB-B and combined XLGB group (**Fig. 1**).

### Analysis of bone formation and resorption markers of XLGB fractions

Table 4 also showed that level of bone formation marker PINP of OVX mice was significantly higher than that of the Sham group (*P*<0.01). XLGB-B and combined XLGB groups showed significantly lower PINP level compared to that of the OVX group (*P*<0.05). Bone resorption marker CTX level in OVX group was higher than that of the Sham group, yet without significance. XLGB-B group had significantly lower CTX level while XLGB-C showed significantly higher CTX level (*P*<0.05 for both, *vs* OVX group).

### Identification of active compounds in XLGB-B fraction

**Fig 2:**
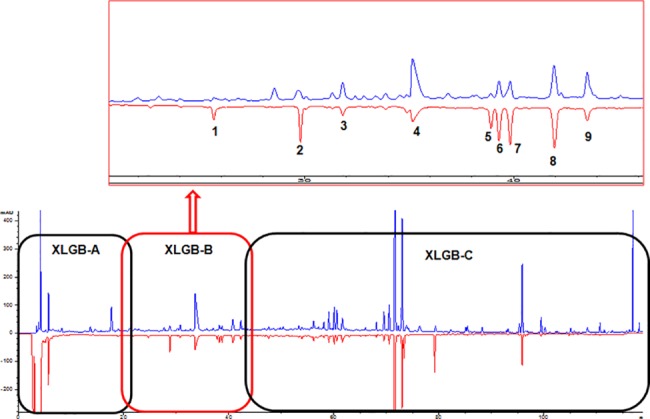
HPLC fingerprint chromatograms of XLGB extract. The blue line is the fingerprint of UV detector and the red line is the one of ELSD. Three fractions (XLGB-A, B, C) were indicated in the square, and the XLGB-B part was amplified in the upper right square. Names of the peaks (1–9) were listed in Table 5.

The peaks of the HPLC fingerprint of XLGB-B identified with the compounds (Fig. 2 and Table 5) isolated were as follows: peak 1 (23.69 min, Tyrosol-1-O-β-xylopyranosyl-(1→6)-O-β-Glucopyranoside); peak 2 (28.6 min, Loganic acid); peak 3 (33.13 min, Chlorogenic acid); peak 4 (35.06 min, Cryptochlorogenic acid); peak 5 (37.32 min, Loganin); peak 6 (38.23 min, Sweroside); peak 7 (38.46 min, Olivil-4-O-β-D-glucopyranoside); peak 8 (42.27 min, Psoralenoside); peak 9 (43.80min, Isopsoralenoside). The sum of the peak areas from peak 1 to 9 occupied 90.0% of the total peak area. According to the structures of the compounds and the literature data of the chemical constituents in the six herbs of XLGB, we identified their sources, with Peak 1, 3, 4, 8 from *Herba Epimedii (*H.E.), 2,5,6 from *Radix Dipsaci* (R.D.), and 8,9 from *Fructus Psoraleae* (F.P.).

**Table 5:** Rention times, names and sources of the nine compounds identified from XLGB-B fraction.

**Peak No.**	**Rt (min)**	**Herb Sources** *	**Name**
1	23.69	H.E.	Tyrosol-1-O-β-xylopyranosyl-(1→6)-O-β-Glucopyranoside
2	28.6	R.D.	Loganic acid
3	33.13	H.E.	Chlorogenic acid
4	35.06	H.E.	Cryptochlorogenic acid
5	37.32	R.D.	Loganin
6	38.23	R.D.	Sweroside
7	38.46	H.E.	Olivil-4-O-β-D-glucopyranoside
8	42.27	F.P.	Psoralenoside
9	43.8	F.P.	Isopsoralenoside

* **H.E.**: Herba Epimedii (淫羊藿); **R.D.**: Radix Dipsaci (续断); **F.P.**: Fructus Psoraleae (补骨脂)

### Promotion of osteogenesis and inhibition of adipogenesis of the identified compounds in XLGB-B fraction *in vitro*

**Fig 3:**
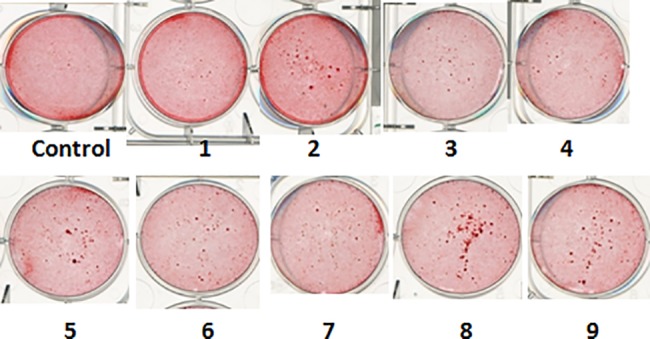
Effect of the nine compounds on the mineralization of UMR 106 cells using Alizarin Red S staining for demonstrating calcium nodules.

Table 6 showed that proliferation of rat osteoblast-like UMR 106 cells was all significantly higher after *in vitro* treatment with Compounds 1, 3, 4 and 7 from *Herba Epimedii*. Compound 2 at 0.01μM and Compound 6 at 1μM from *Radix Dipsaci* also significantly increased cell proliferation (*P*<0.05 for both). While Compounds 8 and 9 from *Fructus Psoraleae* had no effects on UMR 106 cell proliferation at the selected doses. Fig. 3 showed the effect of the nine compounds and the negative control on the mineralization of UMR 106 cells using Alizarin Red S for staining calcium nodules. Compounds 2, 5 and 6 from *Radix Dipsaci* and Compounds 8 and 9 from *Fructus Psoraleae* obviously promoted mineralization. Compounds 5 and 6 from *Radix Dipsaci* and 7 from *Herba Epimedii* significantly inhibited the formation of adipocytes (*P*<0.05 for both).

**Table 6:** Effect of the nine compounds on the proliferation of UMR 106 cells and adipogenesis in 3T3-L1 cells.

Compound	Proliferation of UMR 106 cells			Adipogenesis
	0.01 uM	0.1 uM	1 uM	1 uM
1	0.559±0.0293**	0.5883±0.0296***	0.5363±0.0141	111.06±3.67
2	0.5798±0.0415*	0.5590±0.0446	0.5315±0.0196	112.23±8.21
3	0.5265±0.0212	0.5368±0.0203*	0.5023±0.0216	118.48±2.39
4	0.5578±0.0048***	0.5213±0.0139	0.5033±0.0118	95.64±3.17
5	0.5163±0.0200	0.5205±0.0273	0.5138±0.0369	54.46±1.69**
6	0.5173±0.0215	0.5240±0.0266	0.5595±0.0497*	62.26±4.82**
7	0.6278±0.0578***	0.5583±0.0091*	0.5633±0.0149*	68.71±3.98*
8	0.5370±0.0264	0.5335±0.0232	0.4987±0.0345	81.78±3.92
9	0.5128±0.0158	0.5057±0.0214	0.5250±0.0286	90.96±5.22
Control	0.498±0.0164 (Mean ± SD)			100

Mean value was significantly different from that of the control group

* P<0.05

** P<0.01

*** P<0.001

The proliferation and adipogenesis experiments were performed with at least 3 independent experiments, and the analysis was carried out with triplicate samples.

(Mean values ± standard deviations).

## Discussion

This study was the first one designed to screen the bioactive fraction(s) from a clinical prescribed TCM herbal Fufang Xian Ling Gu Bao (XLGB) formulated with six-herbs using an established *in vivo* mice model. Anti-osteoporosis Fufang XLGB was proven effective and safe on treatment of postmenopausal osteoporosis in an international clinical trial [9] and also it was curative for osteoporosis in OVX rats in a recently published paper where it was used as a positive TCM control[13]. These data formed a foundation for evaluation of its fraction(s) in order to confirm their biological effects on prevention and treatment of OVX-induced osteoporosis and also towards further modification by using less herb materials but with equal or event better treatment efficacy. However, as numerous phytochemicals may co-exist in a herbal formula [32], the sustainable approach is to start with specifying or grouping their fractions together in order to reduce the amount of phytochemicals for *in vivo* screening. Luo et al. mentioned such strategy to study the TCM formula [9,33] by using chromatography to divide the herbal Fufang or formulae into different fractions for identifying bioactive fraction(s) based on their pharmacodynamics, followed by HPLC fingerprint to quantify the fraction and the bioactive compounds for quality control of the herbal drugs. By using this strategy, we have studied the effective material basis of *Drynaria* on preventing osteoporosis [23]. In this study, we divided the XLGB formula into three fractions with different phytochemical constituents using chromatography for *in vivo* screening using OVX-induced osteoporosis mouse model.

### XLGB-B was the bioactive fraction to prevent osteoporosis

The results clearly demonstrated that the 30% ethanol elutes (XLGB-B fraction) of the XLGB extract was the most potential one among the three fractions in prevention of OVX-induced bone loss quantified for their bone mass, bone microarchitecture, bone strength, and bone turnover biochemical markers. Moreover, the present study identified the major components isolated from XLGB-B, i.e. phenylpropanoids from *Herba Epimedii*, terpenes from *Radix Dipsaci* and coumarins from *Fructus Psoraleae*. In addition, the identified compounds effectively promoted proliferation and/or mineralization of osteoblast-like UMR 106 cells, as well as inhibited adipogenesis in preadipocytes 3T3-L1 cells *in vitro*, supporting the material or substance basis of the osteogenic XLGB-B fraction. Most importantly, the treatment dose of XLGB-B (31 mg/kg for mice) tested in the present study for the improvement of bone properties would be equivalent to an oral administration of 2.5 mg/day (the conversion factor from mice to human is 0.081 [25], so the dose for human is 31 mg/kg/day × 0.081 = 2.5 mg/kg/day) in postmenopausal women with a body weight of 60 kg [24], i.e. 60 kg ×2.5 mg/kg/day = 150mg/day, while the doses of XLGB capsule in clinical use was 1.5 g/day [9], implying that the effective doses of XLGB-B to prevent osteoporosis was only one tenth of that of XLGB capsule currently prescribed for osteoporotic patients.

### For preventing bone loss, XLGB-B had similar effects with the M-XLGB and combined XLGB, but less potential than estradiol (E_2_)

In the 6^th^ vertebrae, as compared to OVX alone, middle dose of XLGB extract-treated OVX rats showed 10.2%, 27.6%, 9.2% and 4.1% higher values in BMD, BV/TV, Tb.N, and Tb.Th, respectively; combine-XLGB promoted 7.24% and 9.84% higher in BMD and BV/TV, respectively; while XLGB-B-treated ones showed similar effects, i.e., 9.4%, 16.9%, 9.1%, and 8.6% higher values in BMD, BV/TV, Tb.N, and Tb.Th, espectively, whereas values were 12.4% lower in Tb.Sp. In a recently published study, it showed that XLGB had similar effects, i.e. on BMD and femoral maximum load with Alendronate[13]. However, in our own research XLGB-B was less potent on preventing bone loss than estradiol (E_2_), a prevalently prescribed drug for treatment of osteoporosis. In our other *in vivo* study with the same protocol with this one, estradiol was found to promote 22.22% and 57.14% in BMD and BV/TV respectively. This phenomenon was also found in clinical trials that TCM increased less BMD than HRT, however, TCM has similar or even better effects on preventing bone fracture when comparing with HRT, which might be due to the non-skeletal factors[34,35].

As a phytoestrogen-rich TCM, XLGB did not exert similar effect as estradiol on uterus. Estradiol was reported significantly to induce increase in uterus weight in OVX animals [36], but XLGB fractions did not result in uterus weight in OVX mice (***S1 Fig.***) and rats (***S2 Fig.***). Estrogen expresses its activities by binding to different estrogen receptors (ERs), including ERα and ERβ. ERβ is more abundant than ERα in bone tissue, while ERα it is mainly distributed in reproductive cells and is the dominant receptor mediating the effects of E_2_ in breast and uterus [37]. In our study XLGB-B had anti-osteoporotic effects without affecting body or uterus weight in OVX animals, so it is postulated that the XLGB-B may have potential anti-osteoporotic effects on OVX mice via ERβ in bone or membrane/cytoplasm-initiated estrogen receptor signaling pathway.

### The mainly composed herbs in XLGB-B were well known in strengthening bone

In phytochemical analysis, we identified nine main phytomolecules in XLGB-B fraction from three herbs, namely *Herba epimedii* [38], *Radix dipsaci* [39,40] and *Psoralea corylifolia* L[41,42] **(Table 5).** Interestingly, all these three herbs were tested individually and demonstrated previously for their potential in prevention of osteoporosis. Total flavonoid fraction of the Herb *Epimedii* extract prevented bone loss in OVX mice by increasing renal Ca re-absorption, stimulating the process of osteoblast formation as well as suppressing the process of osteoclastogenesis in OVX mice [43]. *Radix Dipsaci* extract prevented the loss of bone mass, enhanced the bone strength and prevented the deterioration of trabecular microarchitecture in OVX rats [44,45]. P*soralea corylifolia* L. extract and its main compound psoralen were reported to be able to decrease both urinary calcium excretion and serum osteocalcin in OVX rats that resulted in positive effects on maintaining BMD by increasing bone formation rate [46,47]. In coincidence, the other three herbs used for formulating XLGB Fufang, i.e. *Radix salvia miltiorrhizae*, *Rhizoma anemarrhenae* and *Rehmannia glutinosa*, were reported to have biological effects on other tissues instead of bone, e.g. *Radix salvia miltiorrhizae* was used to treat coronary artery disease or other cardiovascular diseases [48], *Rhizoma anemarrhenae* was recorded to ameliorate diabetes-associated cognitive decline in rats [49], and *Rehmannia glutinosa* possesses wide pharmacological actions on the circulation, immune, endocrine, cardiovascular and the neural system [19]. These findings supported that XLGB-B was the most potent fraction for prevention of bone loss as compared among other XLGB fractions.

### XLGB-B prevented osteoporosis by reducing bone turnover rate

Biochemical markers of bone turnover are useful for monitoring the treatment efficacy of anti-osteoporosis drugs in both clinical and preclinical studies [50,51]. PINP is a sensitive bone formation marker in humans [52]. It is the propeptide released from the newly synthesized pre-procollagen prior to the incorporation of collagen molecules into the bone matrix [28]. CTX is a bone resorption marker, which is generated by cathepsin K and released during osteoclastic bone resorption. It is well known that treatment of osteoporosis patients with anti-catabolic compounds decreases the serum level of bone turnover makers, while anabolic treatment increases their serum level [52]. At the early stage of postmenopausal, the bone turnover is up-regulated, i.e. bone formation and bone resorption are both increased while the estrogen level is decreased [52]. In line with our observation in the present study that PINP and CTX were both higher in the OVX group as compared to the Sham-operated mice. Further, our data also showed good correlations between the serum biochemical markers and the bone histomorphometry [51], i.e. BFR/BS was higher in OVX mice than that in the sham-operated ones. In addition, we found that XLGB-B decreased the concentrations of PINP and CTX, as well as BFS/BS as compared to the OVX mice without any treatment, implying that XLGB-B could be regarded a dominant anti-catabolic agent.

### Promoting osteogenesis and inhibiting adipogenesis might be involved in XLGB-B preventing osteoporosis

XLGB-B was mainly composed of nine compounds (Fig. 2 and Table 5). *In vitro* study showed that phenylpropanoids from *Herba Epimedii* had the most potential on increasing the proliferation of osteoblast-like cells than that of other compounds, which was similar to our previous published results on *Drynaria fortunei* [23]. We also found that psoralenoside and isopsoralenoside from *Fructus Psoraleae* significantly promoted the calcium nodules formation of UMR 106 cells without affecting the proliferation of the cells. It was reported that coumarins from *Fructus Psoraleae* stimulated osteogenesis via p38-mediated up-regulation of transcription factors and osteoid genes expression in MC3T3-E1 cells as well as suppression of NFATc1 induction by RANKL [53,54]. Furthermore, inhibition of adipogenesis was found in our study, Compounds 4,5 and 6 suppressed the adipocyte formation *in vitro*, while the lipid accumulation was obviously increased when treatment with XLGB-B (***S3 Fig.***), which reversely promoted osteogenesis. To some extent, the different characters of various types of compounds exert multiple biological effects, which makes multiple-component fraction more effective than single compound in their osteogenic potential for prevention and treatment of osteoporosis [55] and these nine compounds for formulating Fufang XLGB might contribute together to enhance their anti-osteoporosis effects.

### The contribution of XLGB-A and C on preventing osteoporosis

Although, XLGB-B fraction was the most potent one found in the present study on the prevention of bone loss in OVX mice, it did not exclude the contribution of XLGB-A and C in herbal Fufang XLGB. Firstly, they might have other beneficial effects on non-skeletal tissues, organs or body systems, e.g. the polysaccharide in XLGB-A might exert anti-fatigue activity [56] and immunomodulation [19], which were not investigated in this study. Secondly, even we could not exclude the possible potential for preventing bone loss; our chemical analysis showed that most of the *Epimedium* flavonoids known with bone anabolic effects [8,9] were located in the fraction of XLGB-C. However, XLGB-C alone did not show better anti-osteoporosis effects as compared with other two fractions, although both *Epimedium* flavonoids and single compound icariin or icaritin were reported to be effective on preventing bone loss in OVX rats or mice [36,38,43]. It might be attributed to the interactions between *Epimedium* flavonoids and the other phytomolecules from other herbs. Further studies are desirable to explore such interactions among herbal fractions and/or compounds.

## Conclusion

XLGB-B with a yield of 13.0% of herbal Fufang XLGB with clear phytochemical constitutions was screened as the key fraction what demonstrated preventive effects of OVX-induced bone loss in mice. These findings supported an innovative approach towards a new generation of herbal Fufang characterized as “Less herbal raw materials for achieving equal treatment efficacy by traditional herbal formula” on prevention of OVX-induced osteoporosis.

## Supporting Information

S1 Fig All of the XLGB fractions did not affect the uterus weight in OVX mice. The mice were treated for six weeks after operation (n = 10). Mean value was significantly different from that of the OVX group: ** P<0.01.
(DOCX)

S2 Fig XLGB-B did not affect the body weight and uterus weight in OVX rats. The rats were treated for three months after operation (n = 10). Mean value was significantly different from that of the OVX group: * P<0.05, ** P<0.01.
(DOCX)

S3 Fig Representative histological images in Sham, OVX and XLGB-B groups. As it is shown in the following representative H&E stained slices, as compared with the Sham group, ovariectomized mice demonstrate significant number of white lipids in the bone marrow, and XLGB-B however shows obvious less lipids.
(DOCX)
