# Supplementary figures and images for: In Vivo Screening for Anti-Osteoporotic Fraction from Extract of Herbal Formula Xianlinggubao in Ovariectomized Mice
Source: PLoS One. 2015 Feb 19;10(2):e0118184. doi: 10.1371/journal.pone.0118184 (PMC4335011; doi:10.1371/journal.pone.0118184)

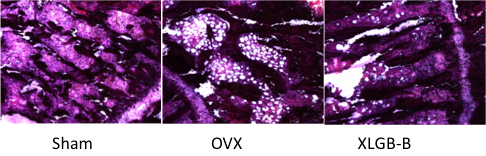

Supplement: S3 Fig — As it is shown in the following representative H&E stained slices, as compared with the Sham group, ovariectomized mice demonstrate significant number of white lipids in the bone marrow, and XLGB-B however shows obvious less lipids. (DOCX) [file pone.0118184.s003.docx]
