# Supplementary material for: In Vivo Screening for Anti-Osteoporotic Fraction from Extract of Herbal Formula Xianlinggubao in Ovariectomized Mice
Source: PLoS One. 2015 Feb 19;10(2):e0118184. doi: 10.1371/journal.pone.0118184 (PMC4335011; doi:10.1371/journal.pone.0118184)
Supplement: Supplementary file 2 [file pone.0118184.s001.docx]

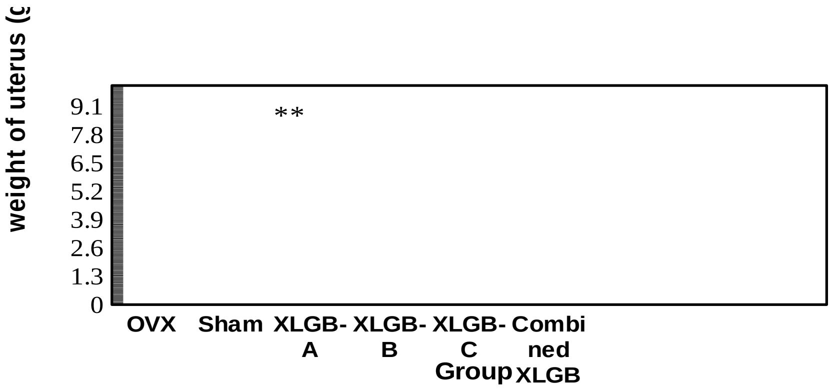

weight of uterus (g
9.1
7.8
6.5
5.2
3.9
2.6
1.3
0
**
OVX
Sham
XLGB-A
XLGB-B
XLGB-C
Combined XLGB
Group
