# Supplementary material for: In Vivo Screening for Anti-Osteoporotic Fraction from Extract of Herbal Formula Xianlinggubao in Ovariectomized Mice
Source: PLoS One. 2015 Feb 19;10(2):e0118184. doi: 10.1371/journal.pone.0118184 (PMC4335011; doi:10.1371/journal.pone.0118184)
Supplement: Supplementary file 3 [file pone.0118184.s002.docx]

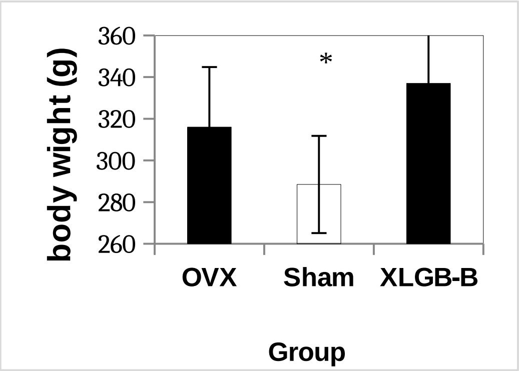
body wight (g)
360
340
320
300
280
260
*
OVX
Sham
XLGB-B
Group

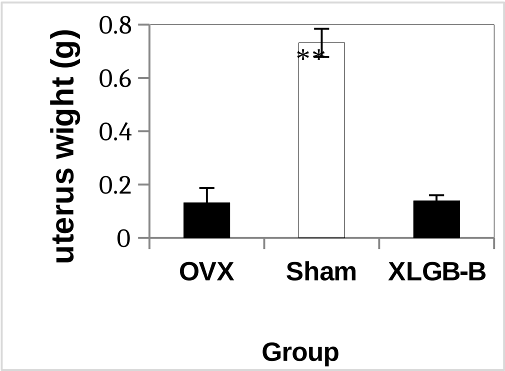
uterus wight (g)
0.8
0.6
0.4
0.2
0
**
OVX
Sham
XLGB-B
Group
